# Conductive Inks Based on Melamine Intercalated Graphene Nanosheets for Inkjet Printed Flexible Electronics

**DOI:** 10.3390/nano12172936

**Published:** 2022-08-25

**Authors:** Magdalena Kralj, Sara Krivačić, Irena Ivanišević, Marko Zubak, Antonio Supina, Marijan Marciuš, Ivan Halasz, Petar Kassal

**Affiliations:** 1 Division of Physical Chemistry, Ruđer Bošković Institute, Bijenička cesta 54, 10000 Zagreb, Croatia; 2 Faculty of Chemical Engineering and Technology, University of Zagreb, Marulićev trg 19, 10000 Zagreb, Croatia; 3 Institute of Physics, Bijenička cesta 46, 10000 Zagreb, Croatia; 4 Division of Materials Chemistry, Ruđer Bošković Institute, Bijenička cesta 54, 10000 Zagreb, Croatia

**Keywords:** mechanochemistry, graphene nanosheets, conductive ink, inkjet printing, printed electronics

## Abstract

With the growing number of flexible electronics applications, environmentally benign ways of mass-producing graphene electronics are sought. In this study, we present a scalable mechanochemical route for the exfoliation of graphite in a planetary ball mill with melamine to form melamine-intercalated graphene nanosheets (M-GNS). M-GNS morphology was evaluated, revealing small particles, down to 14 nm in diameter and 0.4 nm thick. The M-GNS were used as a functional material in the formulation of an inkjet-printable conductive ink, based on green solvents: water, ethanol, and ethylene glycol. The ink satisfied restrictions regarding stability and nanoparticle size; in addition, it was successfully inkjet printed on plastic sheets. Thermal and photonic post-print processing were evaluated as a means of reducing the electrical resistance of the printed features. Minimal sheet resistance values (5 kΩ/sq for 10 printed layers and 626 Ω/sq for 20 printed layers) were obtained on polyimide sheets, after thermal annealing for 1 h at 400 °C and a subsequent single intense pulsed light flash. Lastly, a proof-of-concept simple flexible printed circuit consisting of a battery-powered LED was realized. The demonstrated approach presents an environmentally friendly alternative to mass-producing graphene-based printed flexible electronics.

## 1. Introduction

Flexible electronic devices manufactured by printing techniques on various substrates, such as paper, polymers, and textiles, have recently gained tremendous attention [1,2]. Unlike traditional silicon-based production techniques—often described as costly and complicated—printing offers faster, simpler, as well as environmentally and economically beneficial production possibilities [3,4]. Numerous examples include printed electronic circuits [5], displays [6], radio frequency identification tags (RFIDs) [7], thin-film transistors (TFTs) [8], and sensors [9]. Among different printing techniques, inkjet printing offers several advantages in the publishing and graphics industries [10]. This non-contact additive manufacturing technique is based on the selective ejection of individual drops of a liquid material (ink) from the nozzle upon thermal or pressure pulse [11]; this makes it easily adaptable for mass production. The arrival of inkjet printing outside the scope of classical application came with the development of nanoparticle-based inks with functional properties, especially electrical conductivity [2,12].

Printable inks are based upon a careful selection of the ink components, including the functional material and (a combination of) solvents and stabilizers [13]. Metal nanoparticles [14,15,16], conductive polymers [17,18], and carbon nanomaterials [19,20,21] are the most used functional components in electrically conductive inks. When formulating inks, rheological properties must be carefully tailored to ensure proper jetting. In the case of nanoparticle-based inks, additional restrictions regarding nanoparticle size and ink stability are imposed; these all prevent nozzle clogging.

Even though metal nanoparticles—due to their excellent conductivity—are among the most commonly used materials, a growing interest is being devoted to carbon (nano)materials [22]. Inkjet-printed carbon nanomaterials have therefore been used in the development of flexible and wearable electronics [23,24], sensors [25,26], and film heaters [27]. Considering its intriguing and unique physicochemical properties, such as large surface area, exceptional thermal stability, excellent electrical conductivity, high electron mobility, superior mechanical strength, flexibility, and undemanding chemical functionalization, graphene has attracted attention as a promising functional material in the production of flexible electronic components [28]. To achieve greater concentration in an ink, without the reaggregation of the (nano)particles of the functional material, a suitable solvent based on adequate solubility parameters should be selected; and/or a stabilizing agent should be employed [29,30]. Common solvents for carbon (nano)material-based inks, such as N-methyl-2-pyrrolidone (NMP), N-cyclo-2-pyrrolidone, dimethylformamide (DMF), and dimethylsulfoxide (DMSO), are either expensive, chemically harsh, toxic, and/or difficult to remove post-printing due to their high boiling points [30,31]; thus, their use is not recommended [31,32,33]. However, environmentally compatible solvents often require the additional use of stabilizers, such as polymers and surfactants. For example, graphene suspensions in cyclohexanone and terpineol have been stabilized with ethyl-cellulose [34,35]; in water [36] and ethylene glycol [37] with the stabilizing agent sodium dodecyl sulfate (SDS) [38]; and in ethanol, ethanediol, propanetriol, and deionized water along with sodium carboxymethyl cellulose (CMC) [39].

Different synthetic routes toward graphene have been thoroughly investigated since its discovery. These include the two main approaches: the top-down (TD) approach and the bottom-up (BU) approach [28,32,40,41]. BU approaches, based on the nucleation of a carbon precursor, are generally expensive and time-consuming. On the other hand, in the TD approach, carbonaceous materials (such as graphite) are cut into nano-sized particles by physicochemical processes, which pave the road to the mass production of graphene. TD approaches include the famous Hummers’ method and liquid-phase exfoliation (LPE) of graphite [42,43,44]. Yet, these have major limitations, such as the need for harmful and complex pretreatments, high energy consumption, low yields, agglomeration tendency, low-stability in polar solvents, high precursor costs, or the need for special equipment [41,45].

Clearly, a facile, sustainable, reproducible, and low-cost route for the large-scale preparation of graphene nanosheets (GNS) with minimal surface defects is required to satisfy the growing industry requirements. For this reason, methods of mechanochemistry have become attractive as they often provide quick and quantitative reactions of solids, even on a large scale, while according with the principles of Green Chemistry [46]. There are numerous examples of mechanochemical synthesis and modification of monodisperse nanoparticle systems in a solvent-free environment [47,48,49]. As recently demonstrated, graphite can be exfoliated through non-covalent interactions with melamine (1,3,5-Triazine-2,4,6-triamine) in a ball milling process under solid, i.e., dry conditions [50]; this is in contrast to the exfoliation of graphite with melamine in aqueous media [51,52,53,54].

We present here a facile, scalable, and green method for the development of inkjet printable conductive graphene-based inks. We have adopted a mechanochemical route for the exfoliation of graphite with melamine to form melamine-intercalated graphene nanosheets (M-GNS). The M-GNS were used as the conductive material in the formulation of an inkjet printable ink, with the aid of polymeric dispersants in green solvents (water, ethanol, ethylene glycol). The electrical properties of the printed features were evaluated and post-print processing optimized, to yield flexible printed electronic circuits.

## 2. Materials and Methods

### 2.1. Materials

Graphite flakes (G) having particle sizes 200–300 µm were purchased from Graphenea, San Sebastian, Spain and melamine from Alfa Aesar, Kandel, Germany. Ethanol (absolute) and 2-propanol were obtained from Gram-Mol, Zagreb, Croatia, ethylene glycol from Sigma-Aldrich, St. Louis, MO, USA, and terpineol (mixture of isomers) from Alfa Aesar, Kandel, Germany. All the chemicals were of analytical grade and were used as received. Melamine intercalated graphene nanosheets (M-GNS, 1–2 sheets) were obtained by a mechanochemical route and can be used without additional purification. Aqueous solutions were prepared with deionized water (Millipore Milli-Q, specific conductivity 0.059 μS cm^–1^). Commercial polymeric stabilizing agents Solsperse 12000S and Solsperse 20000 were supplied by Lubrizol, Wickliffe, OH, USA. Surface mount light-emitting diodes for the proof of concept experiment were from Kingbright Electronic Co, New Taipei City, Taiwan; they were glued to the printed conducting traces using a conductive glue, Wire Glue, Anders Products, Melrose, MA, USA.

### 2.2. Mechanochemical Synthesis of Melamine-Intercalated Graphene Nanosheets (M-GNS)

Single- and double-layer melamine-intercalated graphene nanosheets (M-GNS) were obtained by neat grinding in a ball to powder ratio *m_b_*:*m_p_* = 1:12.7. The process was performed at room temperature using a planetary ball mill PULVERISETTE 6 operating at 500 rpm, in a 50 mL stainless steel jar equipped with 12 stainless steel balls (*m* = 4 g; *d* = 10 mm). Graphite flakes (*m* = 0.5 g), melamine (*m* = 2.5 g), and dry ice (*m* = 0.8 g) were milled in a mass ratio *m*(G):*m*(I):*m*(M) = 1:1.6:5 for 48 h in periods of 1 h milling, followed by 10 min of resting. The product was a black free-flowing powder that could be easily collected from the jar using a spatula.

### 2.3. Preparation of M-GNS Inks

M-GNS inks were prepared by dispersing the powdered product in various solvents using a Sonopuls Serie 2000.2 tip-sonicator, with the addition of Solsperse stabilizers. The sonication was performed for 15 min at 25% amplitude of the initial power of 70 W. The physical properties of the as-prepared formulations, including viscosity and surface tension, were measured with a micro-Ostwald viscosimeter 516 13/Ic, SI Analytics GmbH, (Mainz, Germany) and KRÜSS K6 tensiometer (Hamburg, Germany), respectively. All the measurements were performed at room temperature (23 ± 2 °C).

### 2.4. Inkjet Printing of M-GNS Inks and Post-Printing Processing

Inkjet printing was performed using a Fujifilm Dimatix DMP-2850 (Tokyo, Japan) drop-on-demand printer, which utilizes 16 nozzles with a diameter of 21 μm and a nominal drop volume of 10 pL. The experimental printing parameters were optimized to achieve continuous conductive features of the deposited ink on the selected substrates: PI (Kapton, DuPont, Wilmington, NC, USA, *d* = 25 μm); and clear PET (Melinex 505, DuPont, Wilmington, NC, USA, *d* = 125 µm). The printed patterns for characterization were 8 mm × 8 mm squares designed in Dimatix Drop Manager Software 3.0, Fujifilm, Tokyo, Japan.

To improve electrical conductivity, the printed squares were processed both thermally and photothermally using intense pulsed light (IPL). For thermal processing, the specimens were placed in a furnace (Demiterm, Estherm d.o.o., Sveta Nedelja, Croatia) at different temperatures for 1 h. For IPL processing, the jetted patterns were set approximately 1 cm from the flash lamp (Xenon, Wilmington, NC, USA, LH-912) of a Xenon X-1100 IPL system. A series of experiments were performed to find the optimal energy at 2500 V.

### 2.5. Characterization

Powder X-ray diffraction data were collected on a Aeris bench-top diffractometer, Panalytical, Almelo, Netherlands, with Ni-filtered CuK*α* radiation obtained from an X-ray tube operating at 7.5 mA and 40 kV, in the 2*θ* range of 5–70° (step size of 0.027166°, 7.65 s per step). Thermogravimetric measurements were performed with a Shimadzu, Kyoto, Japan, DTG-60H analyzer at a heating rate of 10 °C min^−1^ from room temperature to 1000 °C in a stream of nitrogen, for bulk M-GNS samples and the prepared ink; and from room temperature to 1000 °C in a stream of oxygen for the polymeric stabilizers. Scanning electron microscopy (SEM) imaging was performed on a Jeol, Tokyo, Japan, JSM-7000F field emission scanning electron microscope, operating at 10 kV; while energy-dispersive X-ray spectroscopy (EDX) analysis was performed on an Oxford Instruments, Abingdon, UK, INCA 350 spectrometer coupled with the FE-SEM. Atomic force microscopy (AFM) micrographs were obtained by NanoWizard 4 ULTRA AFM, Bruker, Billerica, MA, USA in AC mode. Samples for AFM were prepared by diluting the stock solutions to a given concentration of 10^−3^ mg/mL and spin coating on a freshly exfoliated mica substrate before drying at 70 or 150 °C in a Biobase Bov-30V Lab high-temperature vacuum oven for 2 h. Fourier-transform infrared attenuated total reflectance (FTIR-ATR) spectra in KBr tablets were recorded on a PerkinElmer, Waltham, MA, USA, SpectrumTwo L1600400 spectrometer equipped with a diamond cell in the range of 4000−450 cm^−1^ with a resolution of 8 cm^−1^. UV–Vis spectroscopy of the conductive ink was performed with a Shimadzu, Kyoto, Japan, UV-1280 UV–Vis spectrometer. The absorption spectra were recorded in the range 320–800 nm after diluting to 1:100 to assure a meaningful absorbance range. Particle size distribution (PSD) was determined using a Zetasizer Ultra (Malvern Panalytical, Malvern, UK) based on a He-Ne laser (*λ* = 632.8 nm) and a thermostated sample cell. The sample dilution was *φ* = 1:33, accounting for the graphene refractive index of 1.957. Before measurement, the sample was equilibrated for 120 s at 25 °C ± 0.1 °C. The intensity of the scattered light was converted into contribution per number of particles within the measured sample volume. Zeta-potential measurements of the M-GNS ink formulation were carried out using the aforementioned instrument and the same thermostated sample cell. The ZS Xplorer v1.00, Malvern Panalytical, Malvern, UK software was used for data analysis. The sheet resistance of the printed samples was measured before and after both thermal and IPL processing using a four-point probe (Ossilla, UK).

## 3. Results and Discussion

### 3.1. Synthesis and Characterization of Melamine-Intercalated Graphene Nanosheets

**Figure 1:**
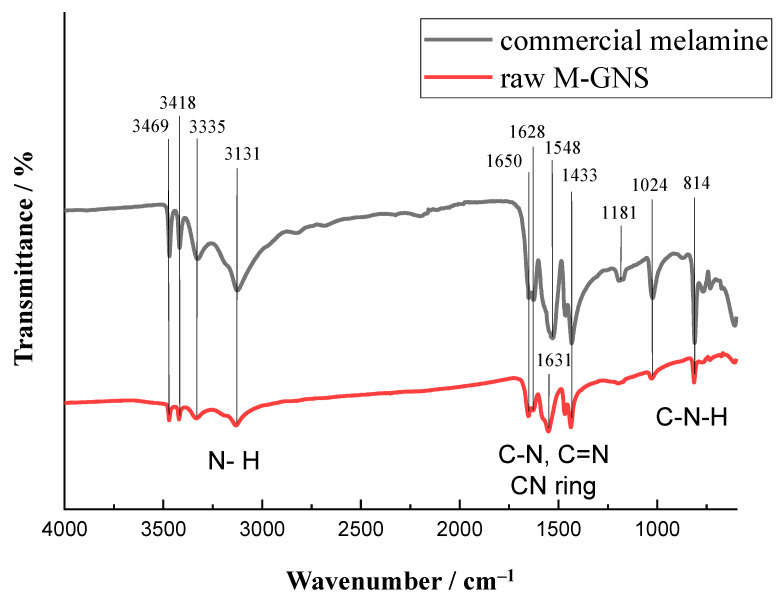
Fourier-transform infrared (FTIR) spectra for the raw sample of melamine-intercalated graphene nanosheets (M-GNS).

For the synthesis of the conducting nanoparticles, we adopted melamine-induced exfoliation in a planetary ball-mill that produces melamine intercalated single and two-layered graphene nanosheets (M-GNS). The role of melamine is to aid the exfoliation of graphite by noncovalent interactions, and prevent re-aggregation of the graphene sheets into a graphitic structure. Melamine has an aromatic core that interacts with the π-system of graphene; however, multiple melamine molecules can form extended 2D networks via hydrogen bonding, and this improves the exfoliation and stabilization of GNS [50]. The synthesized M-GNS were thoroughly characterized to determine their composition, morphology, and thermal properties. The Fourier-transform infrared attenuated total reflectance (FTIR-ATR) spectrum of M-GNS exhibits bands characteristic of melamine, while the absence of any additional bands demonstrates that the sample was pure (Figure 1).

**Figure 2:**
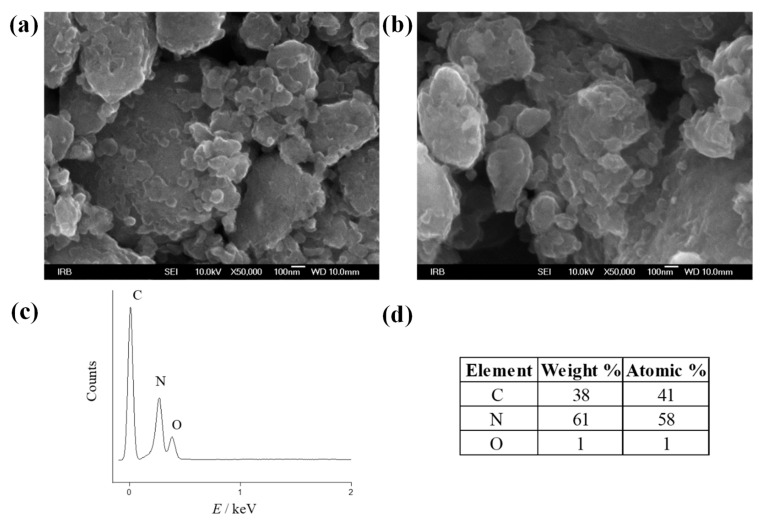
(**a**,**b**) Scanning electron microscopy (SEM) images and (**c**,**d**) energy-dispersive X-ray (EDX) spectra of the raw M-GNS.

Scanning electron microscopy (SEM) was used to evaluate the formation, size distribution, and morphology of the M-GNS; while EDX analysis provided additional information about the elemental composition of the sample. Figure 2 shows the morphology and elemental structure of the raw M-GNS sample. It is noticeable that melamine, after undergoing a grinding process, is present in the sample (evidenced by the significant nitrogen amount). A wide particle size distribution is also observed. The morphology corresponds to previously examined mechanochemically treated carbon materials [55].

**Figure 3:**
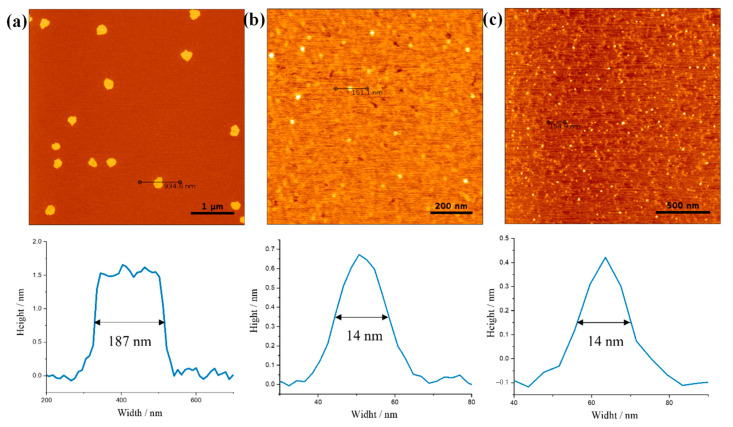
Atomic force microscopy (AFM) images and cross-section analysis of the M-GNS dispersed in a mixture of ethanol:water:EG = 0.50:0.45:0.05. The sample was spin-coated on freshly cleaved mica substrates and was dried in a vacuum oven for 2 h at (**a**) 70 °C and (**b**,**c**) 130 °C.

Topological and morphological studies via atomic force microscopy (AFM) were conducted primarily to visualize the surface structure of the M-GNS and height profiles with and without melamine present in the sample. The AFM image (Figure 3a) shows melamine layers with a lateral dimension of 200 nm. On the other hand, after thermal annealing at 130 °C in a vacuum oven, the larger melamine flakes were removed by sublimation and only smaller graphene nanosheets remained (Figure 3b,c). The average diameter of the GNS was determined to be around 14 nm. AFM height measurements revealed an average height of 0.3–0.65 nm corresponding to single- and double-layer GNS.

The prepared M-GNS were heated from room temperature to 1000 °C to determine their thermal stability. It has been reported that melamine decomposition takes place in three stages, undergoing progressive endothermic condensation during heating; with the release of ammonia and forms products, such as melam, melem, and melon [56]. Products of thermal decomposition of melamine are thermally more stable than melamine. Finally, graphitic carbon nitride, g-C_3_N_4_ is produced under further heating [57,58].

**Figure 4:**
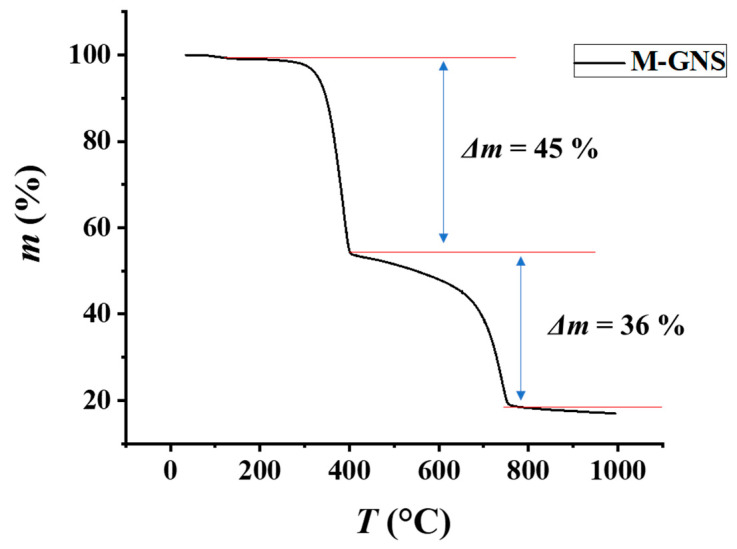
Thermogravimetric analysis (TGA) curve showing the mass loss profile of M-GNS.

As expected, a typical differential weight loss in several regions was observed (Figure 4). The first stage covered the regions of maximum weight loss corresponding to the characteristic mass loss at the range of 300–400 °C. This is associated with melamine condensation to melam (a short-lived intermediate) and further condensation to melem [57]. At higher temperatures (around 400–600 °C), the condensation reaction slowly progresses; at first, it yields melon and then, graphitic carbon nitride [59,60]. Finally, thermal decomposition of graphitic carbon nitride takes place in the range of 600–750 °C [61]; whereas additional changes in weight loss were not observed, proving good thermal stability of GNS. The residual mass of GNS amounts to 18.6%, which is in good agreement with the initial mass ratio of graphite to melamine during the mechanochemical exfoliation.

### 3.2. Preparation and Characterization of M-GNS Inks

**Figure 5:**
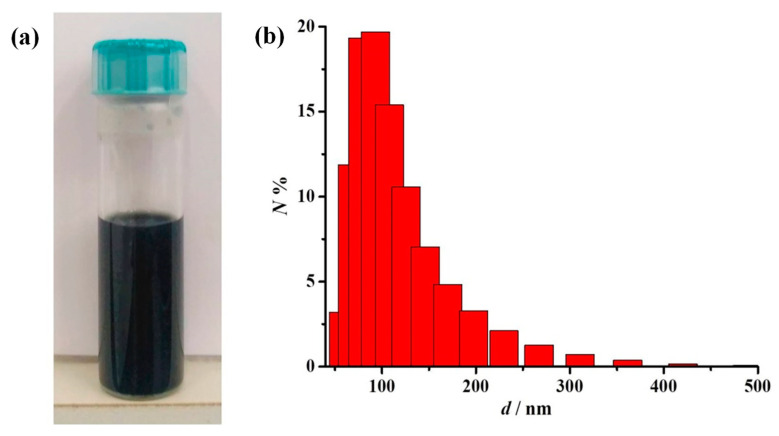
(**a**) The M-GNS ink after 24 h at rest after preparation; and (**b**) a histogram of the prepared M-GNS ink from Dynamic light scattering (DLS) measurements, recorded in an aqueous medium with a sample dilution of *φ* = 1:33.

The M-GNS were dispersed in green solvents using an ultrasonic probe to form conductive inks for inkjet printing. We focused on less harmful polar solvents (water and alcohols) and aimed to formulate an ink compatible with substrates commonly used in printed electronics—PET and PI. Graphene is known to form stable dispersions in solvents with a similar surface energy to itself [62]. When adopting solvents of incompatible surface energy, there is a requirement for stabilizers that adsorb to graphene nanosheets during the homogenization step [36]. In this way, the stability of the ink is improved and the shelf life is significantly extended. Commercially available Solsperse polymeric hyperdispersants—steric stabilizers with anchor groups optimized for strong adsorption to the particle surface—were used for this purpose. We evaluated the stability of the M-GNS in several solvents and their mixtures (see Supplementary Materials, Figure S1). Ultimately, the selected composition of the ink was 2 mg/mL of the M-GNS dispersed in a solvent mixture consisting of ethanol:water:ethylene glycol = 0.50:0.45:0.05 by volume; with the addition of 0.36 mg/mL of Solsperse 20000 and 0.04 mg/mL of Solsperse 12000S stabilizers. The prepared ink is shown in Figure 5a. This composition demonstrated good wetting of PET and PI substrates, with no observable coffee ring effect after drying.

The bottleneck of a piezoelectric drop-on-demand inkjet process is the development of stable, single droplets without the formation of satellite (secondary) droplets [63]. This can be achieved by tuning the inks composition and its physical properties, including viscosity, density, and surface tension. The droplet formation behavior is often characterized by *Z*, a dimensionless inverse Ohnesorge (*Oh*) number [11,64], *Z =* $\sqrt{\gamma \rho a}/\eta$; where *ρ*, *η*, *γ*, and *a* are the density, dynamic viscosity, surface tension, and dimensional parameter of the printer, respectively. Low *Z*-values (<4) indicate possible difficulties in fluid ejection due to the high viscosity, whereas a higher *Z*-value (>13) suggests the formation of satellite droplets or, at least, uncontrollable ink leakage [2]. The requirements for inkjet printable fluids include low viscosity (4–30 mPa s) and relatively high surface tension (around ~35 mN m^−1^) [11]. Our ink formulation had a measured *Z*-value of 7.7, indicating excellent suitability for jetting.

The second major requirement of nanoparticle-based inks is nanoparticle size and suspension stability. A maximum nanoparticle size of about 200–500 nm (1% of the nozzle diameter) is generally suggested, along with the necessary stability against aggregation and sedimentation [12]. Failure to meet either of these requirements can cause the clogging of printer nozzles. Dynamic light scattering (DLS) analysis was performed to determine the particle size distribution of the fabricated M-GNS ink based on the number of scattering particles, Figure 5b.

The histogram in Figure 5b shows that the sample contains particles of different sizes, resulting in an average hydrodynamic particle diameter of *d* = 173.7 nm; this corresponds to the size of melamine sheets observed in the AFM measurements (Figure 3a) and indicates that the nanoparticles are small enough for printing without clogging. The stability of the conductive inks was evaluated using zeta-potential measurements. Particle dispersions with zeta-potential values of ±20–30 mV are generally assumed to be moderately stable [65]. The measured *ζ*-potential of the M-GNS ink was –25.7 mV, indicating moderate stability of the prepared graphene particles in the solution phase [66]. The stability of real systems is determined by the relationship between attractive van der Waals forces (information that is not detectable with *ζ*-potential measurements) and electrostatic repulsive forces between particles (provided by the zeta-potential). Accounting for that, dispersions with a lower absolute zeta-potential than that generally acknowledged should not be discarded in terms of colloidal stability [67].

**Figure 6:**
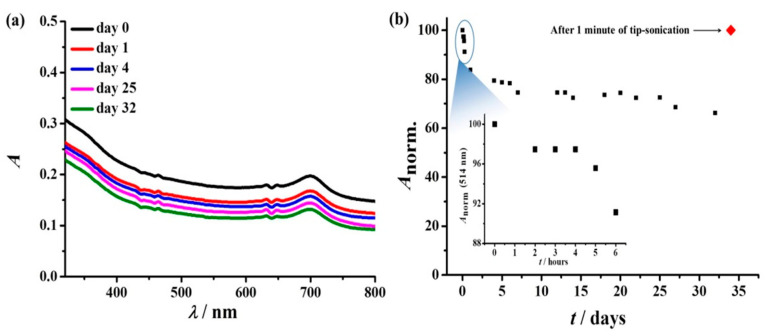
(**a**) A comparison of UV–Vis spectra recorded immediately after ink preparation (**day 0**), and **1, 4, 25** and **32 days** post-preparation (dilution: 100×); (**b**) the normalized view of the absorption at 514 nm (dilution: 100×); (**b**) **inset** sedimentation of the ink within the first six hours after ink preparation.

The long-term stability of the ink was additionally evaluated by optical absorption spectroscopy. We collected UV–Vis spectra of freshly prepared ink and compared it to those taken up to 32 days post-formation. Graphene has an absorbance maximum at ~270 nm [68,69,70]; however, this part of the spectrum is affected by absorption of the Solsperse 20000 stabilizer. Solsperse 12000S, on the other hand, has very strong absorbance in the visible part of the spectrum (Figure S2); while its lowest absorbance is at *λ* = 514 nm. Therefore, 514 nm was chosen as the wavelength for monitoring graphene absorbance reduction as a function of ink instability over time, Figure 6. As can be seen from Figure 6b, sedimentation is strongest within the first 6 h. However, the absorbance at 514 nm does not fall below 91% of the initial value; this indicates good short-term stability for single-day printing. In the following days, the absorbance decreases more slowly; it reaches a minimum at 66% of the initial value after 32 days. On the 34th day of the ink storage, we tip-sonicated the ink for 1 min (25% amplitude) and regained the initial absorbance value (100%). This indicates that although the ink shows moderate stability over prolonged periods, the maximum stability can be recovered after only one minute of ultrasonication.

### 3.3. Inkjet Printing and Post-Printing Processing

**Figure 7:**
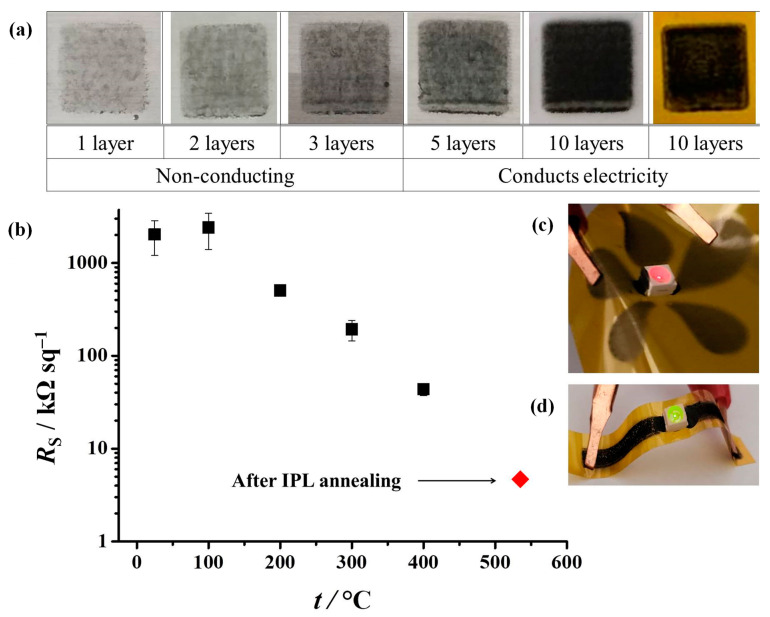
(**a**) The different number of printing passes of the conductive ink on a PET and PI substrate; (**b**) the sheet resistance of printed squares on PI, after thermal annealing at different temperatures. Error bars represent one standard deviation (*n* = 6); red diamond indicates the sheet resistance value after intense pulsed light (IPL) annealing at the energy of 700 J; examples of flexible printed electronics using 10 layers (**c**) and 20 layers (**d**) of the M-GNS ink.

The printing process starts with optimizing the printing parameters, which include the following: waveform, applied voltage, drop spacing, jetting frequency, cartridge height, number of printed layers, cartridge temperature, and platen temperature. The printing was performed at a moderate temperature of 55 °C and low jetting frequency. The other optimized printing parameters are shown in Table S1. For characterization, 8 × 8 mm squares were printed on polyethylene terephthalate (PET) and polyimide (PI) sheets; this is due to these substrates being commonly used in printed electronics [12,71]. Multiple layers of the conductive ink were printed (Figure 7), which is a practical way of increasing conductivity. The printed samples were characterized by sheet resistance measurements (*R*_S_) with a four-point probe. The printed films were not electrically conductive up to three layers. At five layers, the measured sheet resistance was 4.27 ± 0.87 MΩ/sq (SD) and further decreased with additional layers. Nevertheless, such high sheet resistances are inadequate for most printed electronics applications; moreover, increasing the number of printed layers becomes pointless beyond a certain number of layers, since this greatly increases printing duration. Conductivity is instead commonly increased by removal of non-conducting ink components, usually by thermal post-print processing [72].

We exposed the printed squares to thermal annealing in order to improve the electrical conductivity via the removal of melamine and polymeric stabilizers. As presented in Figure 4, most of the melamine thermally decomposes at temperatures of up to 400 °C. The polymeric stabilizer Solsperse 20000 decomposes at somewhat lower temperatures (Figure S4); while Solsperse 12000S is more stable, but present in minuscule amounts. Therefore, the printed squares on PI were processed for 1 h at different temperatures, up to 400 °C (Figure 7b). The sheet resistance decreased gradually with temperature from the initial value of around 2.0 ± 0.9 MΩ/sq (SD), down to 44 ± 6 kΩ/sq (SD) at 400 °C. The thermal processing also benefited the homogeneity of the printed features, as evidenced by the decreasing standard deviations of measured sheet resistances.

**Figure 8:**
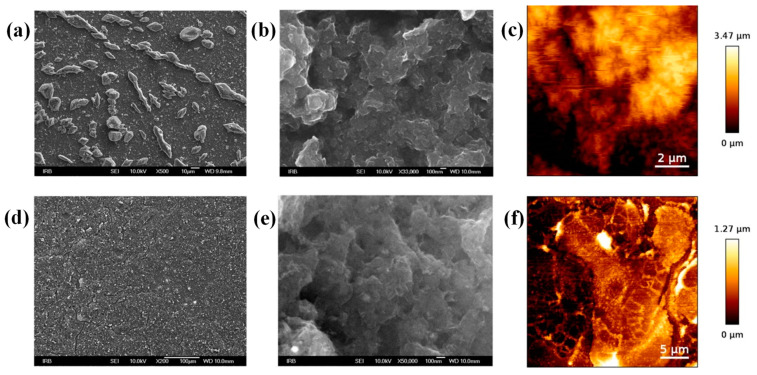
SEM (under different magnification) and AFM images of the M-GNS film on a PI substrate: (**a**–**c**) before annealing; and (**d**–**f**) after annealing at 400 °C.

To gain better insight into the morphology and topology of the printed features, SEM and AFM analysis were performed before and after thermal annealing at 400 °C (Figure 8). The surface morphology of the printed pattern before annealing is rough and inhomogeneous. We observed large melamine crystals (larger than 10 µm in diameter), which disrupt the electrical conductivity (Figure 8a). The SEM picture of the printed pattern after thermal annealing confirms a significant enhancement of the film quality and removal of melamine crystals due to thermal decomposition (Figure 8d,e). Accordingly, AFM measurements revealed a decrease in film thickness after annealing, along with a decrease in surface roughness (Figure 8c,f). The surface roughness parameter *R*_a_ (average) decreased from 580.6 nm to 216.0 nm, while the *R*_q_ (quadratic average) decreased from 789.1 nm to 289.1 nm.

In addition to thermal processing, we evaluated intense pulsed light (IPL) as a way of photothermal processing. IPL uses very short high-energy pulses of visible light; it thereby diminishes the thermal stress on the sensitive polymeric substrate, which makes it highly compatible with printed flexible electronics [73]. Graphene-based materials are great candidates for IPL annealing due to their high absorption coefficient in the visible part of the spectrum [74,75]. Nevertheless, the sheet resistance of printed squares was reduced only to around 43% of the initial value after exposure to 600 J (Figure S5). A further increase in IPL energy caused an increase in resistance, suggesting that the conductive film was damaged during the annealing process. This can be attributed to the formation of gaseous products (ammonia) of melamine decomposition [58] in very short time intervals, leading to the removal of graphene from the substrate. Therefore, IPL in itself is not an optimal processing technology for this kind of conducting ink containing melamine. However, we exposed the previously thermally annealed samples (at 400 °C) to IPL energies of 700 J. This combined processing procedure resulted in the lowest sheet resistances of only 5.0 ± 0.3 kΩ/sq (SD) for 10 printed layers (shown in Figure 7b) and 626 ± 106 Ω/sq (SD) for 20 printed layers. As can be seen from Table S2, the measured sheet resistances are comparable to, or better than, those obtained in similar studies and for a comparable number of printing passes. While printed metal nanoparticle inks can yield the lowest sheet resistances, in some cases less than 1 Ω/sq [12], in the case of graphene inks, sheet resistances are usually larger than 1 kΩ/sq for a single digit number of printed layers. Increasing the number of printing passes reduces the sheet resistance below the value of 1 kΩ/sq, which is usually observed at 20 passes or more (Table S2). Such resistivities are sufficient for different printed electronics applications [23]. Finally, as a proof-of-concept experiment, we constructed simple flexible LED circuits; we constructed the circuits by printing 10 and 20 layers of the conductive ink on PI, either as plain 2 mm wide conducting traces or in the shape of our institution logo (Figure 7c,d). The printed traces were annealed in the same way as previously optimized, by combining thermal and IPL processing. The attached surface mount LEDs were successfully powered from a single 9 V battery.

## 4. Conclusions

We described here a novel mechanochemical synthesis of melamine-intercalated graphene nanosheets; we suggest it as a potential approach for large-scale preparation of graphene, which would comply with the basic principles of green chemistry. The prepared M-GNS were used as a functional material for the formulation of a stable graphene-based ink, suitable for inkjet printing. The printing process was optimized to generate electrically conductive patterns on flexible PET and PI substrates. A combination of thermal and photonic (IPL) annealing reduced the electrical resistance of the printed patterns by three orders of magnitude. The presented procedure is both scalable and environmentally friendly; in addition, it represents a starting point in the development of graphene-based printed flexible electronics.

## Data Availability

The data presented in this study are available in the article and supplementary material.

## Supplementary Materials

The following supporting information can be downloaded at: https://www.mdpi.com/article/10.3390/nano12172936/s1, Figure S1: Stability in different solvents; Figure S2: Absorption spectra of stabilizers and ink formulation; Figure S3: Unsuccessful printing example; Figure S4: Thermogravimetric analysis; Figure S5: Effect of intense pulsed light on sheet resistance; Table S1: Optimized printing parameters; Table S2: An overview of relevant literature. References [31,35,36,37,39,76,77,78,79,80] were cited in Supplementary Materials.
