# Supplementary material for: Conductive Inks Based on Melamine Intercalated Graphene Nanosheets for Inkjet Printed Flexible Electronics
Source: Nanomaterials (Basel). 2022 Aug 25;12(17):2936. doi: 10.3390/nano12172936 (PMC9457697; doi:10.3390/nano12172936)
Supplement: Supplementary file 1 [file nanomaterials-12-02936-s001.zip › nanomaterials-1861375-supplementary.pdf]

S1. Preparation and Characterization of M–GNS Inks

Stability of the M-GNS was tested in several solvents: redistilled water (with and without stabilizers), ethanol (96 wt.%) (with and without stabilizers), terpineol (without stabilizers), as well as their mixtures. The stability of such formulations was visually examined. As shown in Figure S1., the stability against the reaggregation and particle setting is very low for pure water and terpineol without stabilizers. The selected Solsperse stabilizers can be used with polar (e.g. water and alcohol) solvents and they were not soluble in terpineol so all formulations with terpineol were discarded. Generally, stability of the ink formulation decreased upon a decrease in the used solvent polarity. While purely water-based inks with stabilizers are stable, their surface tension is too high for successful printing and wetting of the substrate. On the other hand, purely ethanol-based inks evaporate during printing (due to their low boiling points) which caused nozzle clogging and print failure. Ultimately, a formulation consisting of EtOH:H_2O:EG = 0.50:0.45:0.05 by volume was selected based on the ink stability and rheological properties. The droplet formation behavior can be roughly predicted by the dimensionless parameter Z, which is defined in the main text. The determined physical properties of the printed M-GNS ink include: surface tension, γ = 31.67 mN/m; viscosity, η = 3.26 mPa s; and density, ρ = 0.9373 g/mL. Thus, the calculated Z-value corresponds to 7.7. Absorbance spectra of different ink components are given in Figure S2.

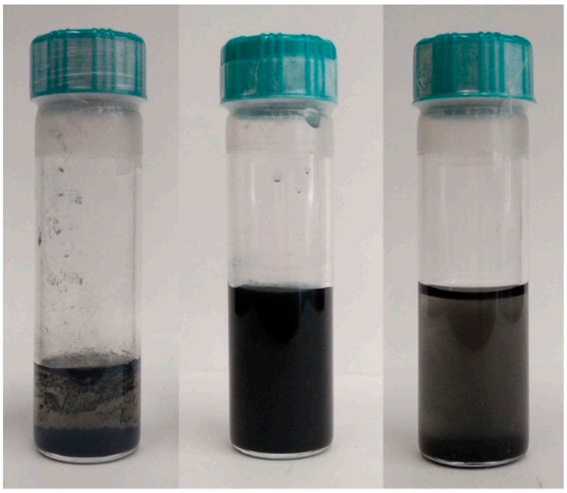

a) b) c)

Figure S1. a) Water-based formulation without stabilizers (γ(MGNs) = 3 mg/mL), 6 days after preparation; **b**) water-based formulation with stabilizers (γ(MGNs) = 3 mg/mL), 7 days after preparation; **c**) terpineol-based formulation without stabilizers (γ(MGNs) = 1 mg/mL), 5 days after preparation.

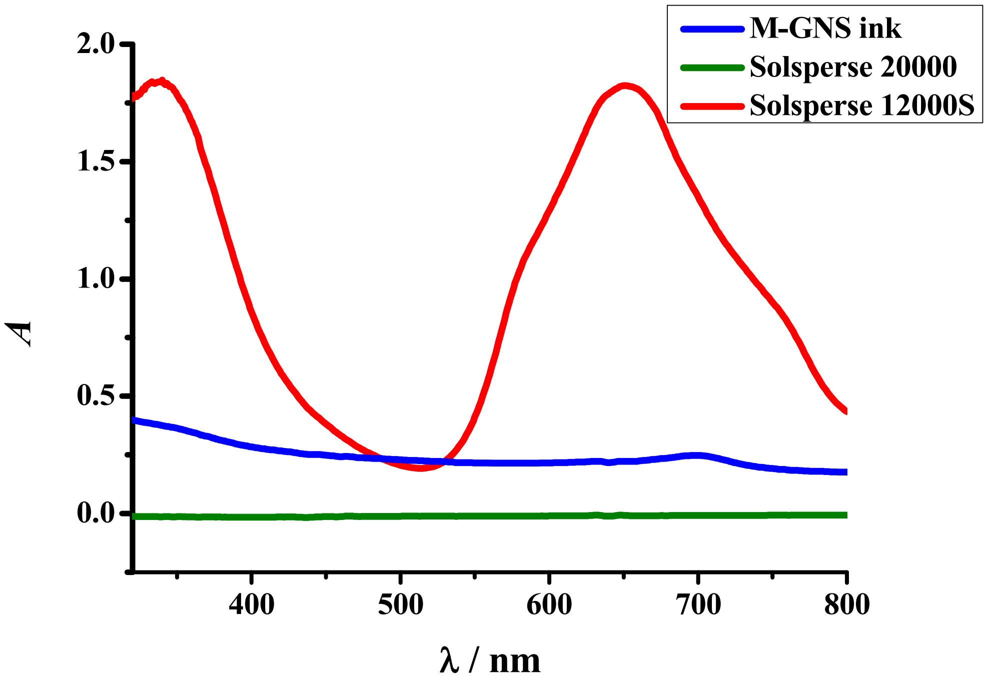

Figure S2. Absorption spectra of stabilizers and ink formulation. Both stabilizers were measured at the concentration as in M–GNS ink; the ink formulation was diluted 100 times.

S2. Inkjet Printing and Post–Print Processing

By determining an appropriate waveform and voltage, the jetting of a low viscosity ink was successfully performed. The printing resolution, determined by the drop spacing (DS), is governed by the overlapping of drops on the substrate. The DS optimization aims to enable just enough overlap to form the conductive lines, i.e. to avoid severe overlapping that could cause merging of the droplets, slower evaporation of the solvent, and consequently, the appearance of the coffee ring effect [1] and inhomogeneous deposits. The ethanol-water-EG based ink is suitable for low energy consuming processes firstly, due to its low viscosity, cartridge heating is completely unnecessary, and secondly, the ink's relatively low boiling point minimizes the prerequisite for high temperature platen heating. All printing parameters are given in Table S1. Unsuccessful printing of an IPA : H_2O based formulation, which did not show adequate wetting of the substrate is shown in Figure S3, for reference.

Table S1. The optimized printing parameters of M-GNS ink on the PET and PI substrates.

**Voltage**	9 V
**Frequency**	10 kHz
**DS**	5 µm
**Cartridge height**	1 mm
**Cartridge temperature**	32.5 °C
**Platen temperature**	55 °C
**Number of overprints**	10

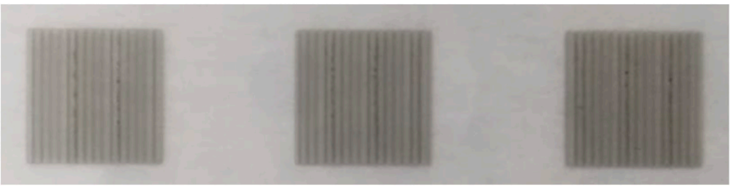

Figure S3. The printed patterns of IPA : H2O based formulation (γ(MGNs) = 3 mg/mL).

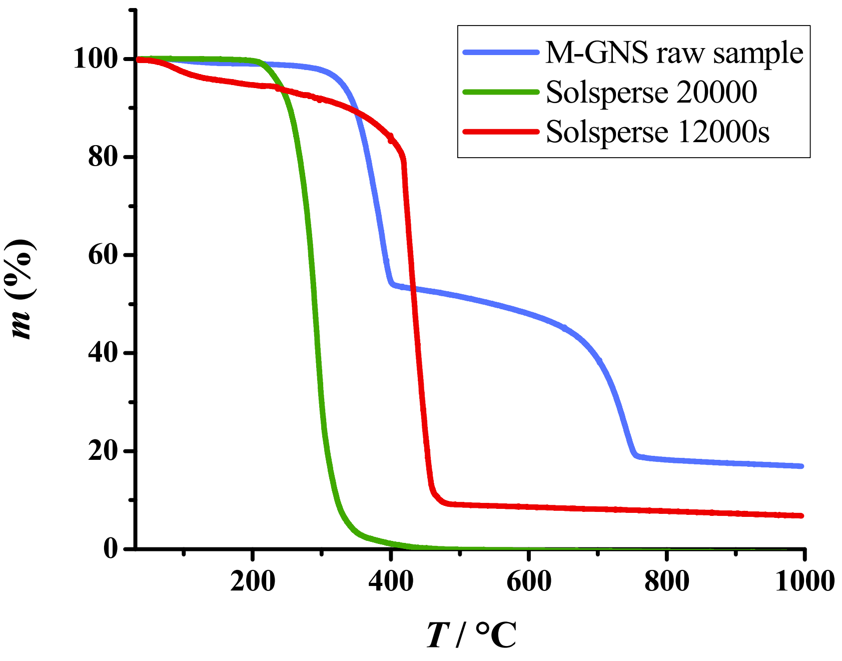

Figure S4. Thermogravimetric analysis (TGA) curve showing the mass loss profile of M-GNS (blue), and hyperdispersants Solsperse 20,000 (green) and Solsperse 12,000S (red).

Complete thermal decomposition of Solsperse 20,000 was observed in the range 180–450 °C. In addition, SOLSPERSE 12,000S decomposition also takes place in two stages. The first stage of the decomposition takes place from room temperature to 180 °C and is associated with moisture removal, mainly associated with evaporation of water content that is present in SOLSPERSE 12,000S. The second stage was observed in the temperature range 180–490 °C, corresponding to the complete decomposition of the stabilizing agent.

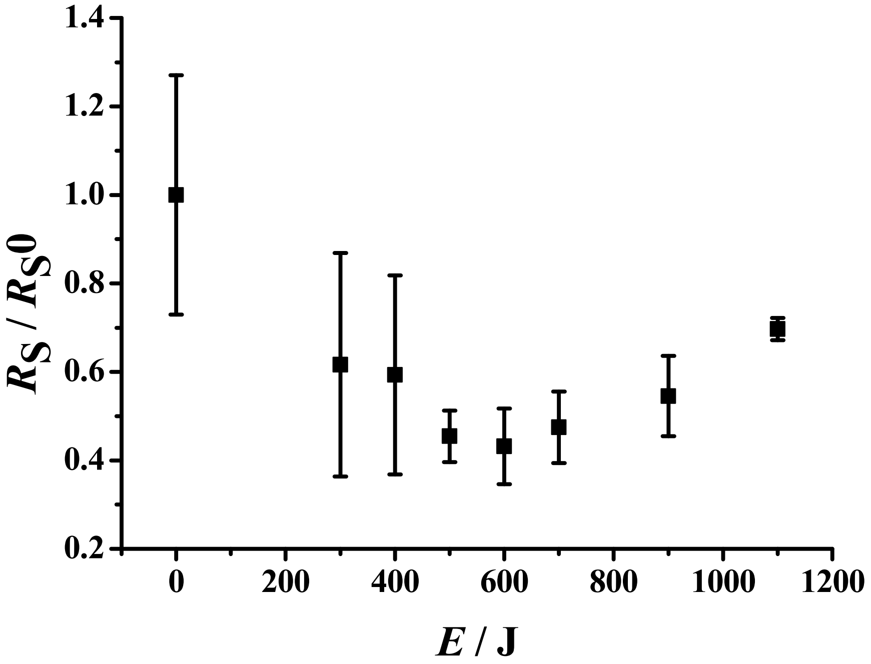

Figure S5. The normalized sheet resistance of printed 8 × 8 mm squares upon irradiation by IPL of different energies at 2500 V lamp voltage. Error bars represent one standard deviation (*n* = 6).

Table S2. An overview of relevant literature in the period from 2015 up to date. GO, Graphene oxide; RGO, Reduced graphene oxide; CB, Carbon black; M-GNS, Melamine-intercalated graphene nanosheets; MLG, Multilayered graphene; EG, Ethylene glycol; EtOH, Ethanol; D-H2O, Deionized water; NMP, N-Methyl-2-pyrrolidone; PG, Propylene glycol; IPA, Isopropyl alcohol, MEG, mono-ethylene glycol; PET, Poly(ethylene terephtalat); PI, Polyimide.

Ref.	Nanomaterial	Concentration of Nanomaterial	Solvent	Number of Layers	Substrate	Processing	Resistance
[39]	RGO and CB	4.31 mg/mL	EtOH, ethanediol, propanetriol, D-H_2O	4	glossy photo paper	thermal annealing at 100 °C, for 30 min	20 kΩ/□
[31]	graphene	3.32 mg/mL	NMP	6	PET	not specified	173 kΩ/□
[76]	graphene	20 mg/mL	85% cyclohexanone; 15% terpineol	8	PET foils	IPL	25 Ω/□
[37]	graphene	3.2 mg/mL	EG	10	plastic foil	thermal annealing ~ 350 ºC for 150 min	260 Ω/□
[77]	GO	5 mg/mL	H_2O:EG = 1:1	10	photo paper	plasma treatment for 120 min	2 kΩ/□
**this work**	**M-GNS**	**2 mg/mL**	**EtOH:H_2O:EG = 50:45:5**	**10**	**PI**	**thermal annealing and IPL**	**5 kΩ/□**
[31]	graphene	0.62 mg/mL	EtOH:H_2O = 1:1	12	PET	not specified	75 kΩ/□
**this work**	**M-GNS**	**2 mg/mL**		**20**	**PI**		**726.01 Ω/□**

			**EtOH:H_2O:EG = 50:45:5**			**thermal annealing and IPL**	
[78]	GO	4 mg/mL	D-H_2O:EtOH:EG = 1:1:1	20	PET	IPL	760.4 Ω/□
[35]	MLG	3.5 mg mL^{-1}	85% cyclohexanone; 15% terpineol	25	PI sheets	thermal annealing at 250 and 350 °C	1.60 kΩ/sq
[35]	MLG	3.5 mg mL^{-1}	85% cyclohexanone; 15% terpineol	30	PI sheets	thermal annealing at 250 and 350 °C	0.89 kΩ/sq
[79]	graphene	3 mg/mL	H_2O:PG=10:1	40	paper	thermal annealing	1 kΩ/□
[36]	graphene	2.25 mg mL^{-1}	H_2O	50	PEL paper	vacuum thermal annealing at 100 °C	1.2 ± 0.2 kΩ/sq
[80]	graphene	0.3 mg/mL	D-H_2O, IPA, MEG	60	photo paper	250 °C, 9 h	266.67 Ω/□

References

31 Capasso, A.; Castillo, A.D.R.; Sun, H.; Ansaldo, A.; Pellegrini, V.; Bonaccorso, F. Ink-jet printing of graphene for flexible electronics: An environmentally-friendly approach. *Solid State Commun.* **2015**, *224*, 53–63. https://doi.org/10.1016/j.ssc.2015.08.011.

35 Pandhi, T.; Cornwell, C.; Fujimoto, K.; Barnes, P.; Cox, J.; Xiong, H.; Davis, P.H.; Subbaraman, H.; Koehne, J.E.; Estrada, D. Fully inkjet-printed multilayered graphene-based flexible electrodes for repeatable electrochemical response. *Rsc Adv.* **2020**, *10*, 38205–38219. https://doi.org/10.1039/d0ra04786d.

36 Parvez, K.; Worsley, R.; Alieva, A.; Felten, A.; Casiraghi, C. Water-based and inkjet printable inks made by electrochemically exfoliated graphene. *Carbon* **2019**, *149*, 213–221. https://doi.org/10.1016/j.carbon.2019.04.047.

37 Majee, S.; Song, M.; Zhang, S.-L.; Zhang, Z.-B. Scalable inkjet printing of shear-exfoliated graphene transparent conductive films. *Carbon* **2016**, *102*, 51–57. https://doi.org/10.1016/j.carbon.2016.02.013.

39 Ji, A.; Chen, Y.; Wang, X.; Xu, C. Inkjet printed flexible electronics on paper substrate with reduced graphene oxide/carbon black ink. *J. Mater. Sci.-Mater. Electron.* **2018**, *29*, 13032–13042. https://doi.org/10.1007/s10854-018-9425-1.

76 Secor, E.B.; Ahn, B.Y.; Gao, T.Z.; Lewis, J.A.; Hersam, M.C. Rapid and Versatile Photonic annealing of Graphene Inks for Flexible Printed Electronics. *Adv. Mat.* **2015**, *27*, 6683. https://doi.org/10.1002/adma.201502866

77 Sui, Y.K.; Hess-Dunning, A.; Wei, P.; Pentzer, E.; Sankaran, R.M.; Zorman, C.A. Electrically Conductive, Reduced Graphene Oxide Structures Fabricated by Inkjet Printing and Low Temperature Plasma Reduction. *Adv. Mat. Tech.* **2019,** *4*, 1900834**.** https://doi.org/10.1002/admt.201900834.

78 Pei, L.M.; Li, Y.F. Rapid and efficient intense pulsed light reduction of graphene oxide inks for flexible printed electronics. *RSC Adv.*, **2017**, *7*, 51711–51720. https://doi.org/10.1039/C7RA10416B

79 McManus, D.; Vranic, S.; Withers, F.; Sanchez-Romaguera, V.; Macucci, M.; Yang, H.; Sorrentino, R.; Parvez, K. Son, S.-K.; Iannaccone, G.; et al. Water-based and biocompatible 2D crystal inks for all-inkjet-printed heterostructures. *Nature Nanotech.* **2017**, *12*, 343–350. https://doi.org/10.1038/nnano.2016.281.

80 Romagnoli, M.; Lassinantti Gualtieri, M.; Cannio, M.; Barbieri, F.; Giovanardi, R. Preparation of an aqueous graphitic ink for thermal drop-on-demand inkjet printing. *Mat. Chem. Phys.* **2016**, *182*, 263–271. https://doi.org/10.1016/j.matchem-phys.2016.07.031.
